# The Values of Potentially Toxic Elements (PTEs) in Prescription and Non-prescription Dry Cat and Dog Diets in Turkey

**DOI:** 10.1007/s12011-025-04680-4

**Published:** 2025-05-30

**Authors:** Bengü Bilgiç, Duygu Tarhan, Fatma Ateş, Gerta Dhamo, Lora Koenhemsi, Banu Dokuzeylül, M. Erman Or

**Affiliations:** 1 Department of Internal Medicine, Faculty of Veterinary Medicine, Istanbul University-Cerrahpasa, Istanbul, Türkiye; 2 Department of Biophysics, School of Medicine, Bahcesehir University, Goztepe, Istanbul 34734 Türkiye; 3 Department of Biophysics, School of Medicine, Bezmialem Vakif University, Istanbul, Türkiye; 4 Faculty of Veterinary Medicine, Agricultural University of Tirana, Kodër Kamëz, 1029 Tirana, Albania

**Keywords:** Toxic elements, Nutrition, Pet food, Diets, Prescription

## Abstract

Prolonged exposure to high doses of certain toxic metals can cause cytotoxic, genotoxic, and carcinogenic effects in cats and dogs. This study aimed to determine the levels of potentially toxic elements in various prescription and non-prescription commercial diets for cats and dogs. A total of 84 dry cat diets and 152 dry dog diets were analyzed. Prescription cat diets were subgrouped into digestive (*n* = 24) and urinary (*n* = 20), while prescription dog diets were categorized as digestive (*n* = 28), urinary (*n* = 16), hypoallergenic (*n* = 20), and joint (*n* = 12). Additionally, non-prescription diets from various brands and flavors were included for dogs (*n* = 76) and cats (*n* = 40). Chromium (Cr), arsenic (As), boron (B), aluminum (Al), and cobalt (Co) concentrations were determined using inductively coupled plasma with optical emission spectrometry (ICP OES) (Thermo iCAP 6000 series) at appropriate wavelengths. No significant differences were found in mean As, B, and Co levels between total dog and cat diets (*p* > 0.05). However, mean Cr and Al levels were significantly higher in dog diets compared to cat diets (*p* < 0.001). Among cat diets, no significant differences were observed for Cr, As, B, Al, or Co (*p* > 0.05). In non-prescription dog diets, mean Cr was significantly higher than in urinary group (*p* < 0.001). Mean Al levels in digestive, joint, and non-prescription groups were higher than in urinary group (*p* < 0.001). The levels of Cr, As, B, Al, and Co in both prescription and non-prescription diets were below the maximum tolerable limits established by FEDIAF, AAFCO, and FDA, indicating no risk of diet-related toxicosis in cats and dogs.

## Introduction

In recent years, the pet diet industry has been growing globally. Balanced, palatable, complete, and nutritionally adequate formulations have been suggested for cat and dog nutrition [1, 2]. These products are specifically formulated for ease of use and convenience. It has been proposed that the extended lifespan of companion animals may be partially linked to advancements in nutritional guidelines and the availability of commercially produced, complete, and balanced diets [3]. A wide range of commercial diets specifically formulated to address the unique nutritional requirements of pets is available on the market as both prescription and non-prescription. The elemental composition of these diets varies significantly [4, 5]. European legislation reported undesirable substances in animal feed establishes maximum allowable levels for various contaminants, including various toxic elements. This regulatory framework seeks to standardize market placement and feed usage conditions, ensuring high feed safety standards and, consequently, safeguarding public health. However, despite the presence of such regulations, enforcement efforts appear to be primarily directed toward feed for production animals rather than pet diets [6].

Low and high concentrations of elements may cause serious health problems in companion animals [7]. Among these elements, chromium (Cr) is a naturally occurring metallic element found in the environment in different forms. High doses and prolonged exposure can lead to various cytotoxic, genotoxic, and carcinogenic, effects in both humans and animals [8–10]. Arsenic (As), as a metalloid, is associated with both acute and chronic toxic effects on the cardiovascular, hematopoietic, nervous, digestive, and urinary systems in various chemical forms and oxidation states [11]. In both animals and humans, natural boron (B) predominantly exists as boric acid which interacts with amino acids, hydroxy acids, carbohydrates, nucleotides, and vitamins, forming molecular additive compounds through electron donor–acceptor interactions at intracellular pH [12]. Although boron plays an essential role on growth performance, bone and embryonic development, liver functions, redox balance, inflammatory processes, and brain activity, high doses were reported as lethal [13]. Aluminum (Al), one of the most common element in the Earth’s crust, has critical neurotoxic effects on humans and animals [14]. Cobalt (Co) has been known as an essential micronutrient in the form of vitamin B_12_ (hydroxocobalamin). However, Co exhibits acute toxicity at high doses, while prolonged exposure, even at low levels, can lead to toxic and carcinogenic effects on multiple organs and tissues, including the thyroid gland, lungs, skin, and the immune system [15].

Numerous studies conducted in various countries have been published in the literature regarding the determination of toxic metal levels in dry pet foods for cats and dogs. A recent study in China reported that, in some pet food samples, the limit values for lead (Pb) (exceeding the upper limit by 8.89% in cat foods and 8.33% in dog foods) and chromium (Cr) (exceeding the upper limit by 22.22% in cat foods and 31.25% in dog foods) as defined by the Hygienical Standard for Pet Feed of China (Announcement No. 20, 2018) were surpassed, while concentrations of other toxic metals (Cd, Hg, As) remained within acceptable limits [16]. In a study conducted in Brazil, the percentage of dog food samples exceeding the maximum tolerable limits (MTL) set by the FDA and FEDIAF for Al, mercury (Hg), lead (Pb), uranium (U), and vanadium (V) were reported as follows: Al, 31.9%; mercury, 100%; lead, 80.55%; uranium, 95.83%; and vanadium, 75%. In cat foods, the corresponding values were as follows: Al, 10.71%; Hg, 100%; Pb, 32.14%; U, 85.71%; and V, 28.57% [17]. In contrast, a study conducted in Poland indicated that, considering the FEDIAF and AAFCO nutritional guidelines, the heavy metal content (Pb, Co, Cd, Cr, Ni) in dry dog foods did not pose any risk to dogs [18].

The critical health effects of these potentially toxic elements (PTEs) highlight the significance of not only environmental exposure but also dietary intake levels. This study aimed to determine the levels of Cr, As, B, Al, and Co in various prescription and non-prescription dry pet diets for cats and dogs and to compare the obtained concentrations between different diet formulations.

## Materials-Methods

### Diet Samples

A total of 84 dry cat diets and 152 dry dog diets collected between 2021 and 2022 were included in the study. Commercial dry cat diets were subgrouped as prescription (digestive (*n* = 24) and urinary (*n* = 20)) and non-prescription (*n* = 40); commercial dry dog diets were subgrouped as prescription (digestive (*n* = 28), urinary (*n* = 16), hypoallergenic (*n* = 20) and joint (*n* = 12)) and non-prescription (*n* = 76). Non-prescription dry cat diets of various brands and flavors, available in the market, as well as prescription dry cat diets of different brands and flavors, exclusively sold in veterinary clinics, were collected for analysis. All pet diet samples were obtained as unopened packages directly from the companies by the researchers using their own resources and were stored under appropriate conditions. Expired samples were not included in the study.

Prescription diet groups were from different companies, flavors, and ingredients manufactured in Italy, France, and Spain. The diets formulated for nutritional support in cases of gastrointestinal diseases, maldigestion, absorptive disorders, diet-related adverse gastrointestinal reactions, weight management, and some pancreatic and hepatic diseases were included in the digestive group. The diets formulated for urinary system support were included in the urinary group. The diets formulated for nutritional support in cases of atopic/allergic dermatitis, diet-related adverse skin reactions, and nutritional intolerances were included in the hypoallergenic group. The diets formulated for joint, bone, cartilage, and mobility support were included in the joint group. Similarly, the non-prescription group included products from different companies with various flavors and ingredients, manufactured in Italy, France, Spain, and Turkey. As protein sources, the pet diets contained dehydrated poultry proteins, hydrolyzed pork, poultry, fish, and various vegetable proteins.

### Determination of Element Concentrations

The concentrations of Cr, As, B, Al, and Co were analyzed in all diet samples using an inductively coupled plasma-optical emission spectrometer (ICP OES; Thermo iCAP 6000 series). Appropriate wavelengths were selected for the quantitative determination of each element. The selected wavelengths were 267.716 nm for Cr, 189.042 nm for As, 249.773 nm for B, 167.079 nm for Al, and 228.616 nm for Co. To ensure a representative sampling, multiple batches of each type of pet diet were collected. From these batches, three subsamples were prepared for each analysis, and the mean concentration values were calculated. The digestion process was performed by adding 2 mL of concentrated nitric acid (HNO_3_ 65% (m/v); Merck) to the diet samples, followed by thermal decomposition in an oven at 200 °C for 20 min. Subsequently, 1 mL of concentrated perchloric acid (HClO_4_; 60% (m/v); Panreac) was introduced into the nitric acid-digested samples and decomposed at 200 °C for 20 min. Approximately 0.2 g of dried sample was used for each digestion. After cooling to room temperature, the samples were vortexed to ensure homogeneity. Although no mechanical homogenization was applied before acid addition, the samples became homogenous during digestion due to complete breakdown of the organic matrix. Following digestion, distilled water was added to adjust the final volume to 10 mL. The samples were vortexed again prior to ICP OES analysis [19–21]. Calibration was conducted using three-point standard solutions and blank solutions, which served as reference points. The calibration curves exhibited high consistency and linearity, with correlation coefficients which were determined for each analyzed element. The ICP OES instrument was operated under the following conditions: plasma gas flow rate of 15 L/min, argon gas flow rate of 0.5 L/min, sample flow rate of 1.51 L/min, peristaltic pump speed of 100 rpm, and RF power set at 1150 W. Each measurement was conducted in triplicate, and the average values were reported. Trace element concentrations in the pet diet samples were expressed in µg/g dry matter.

### Statistical Analysis

Statistical analyses were carried out using the SPSS software package (version 25; SPSS, Inc., Chicago, IL, USA). Kruskal–Wallis and Mann–Whitney *U* tests were used to compare the means. Results were expressed as the mean ± standard deviation (SD), with a significance level of *p* < 0.05.

## Results

**Fig. 1:**
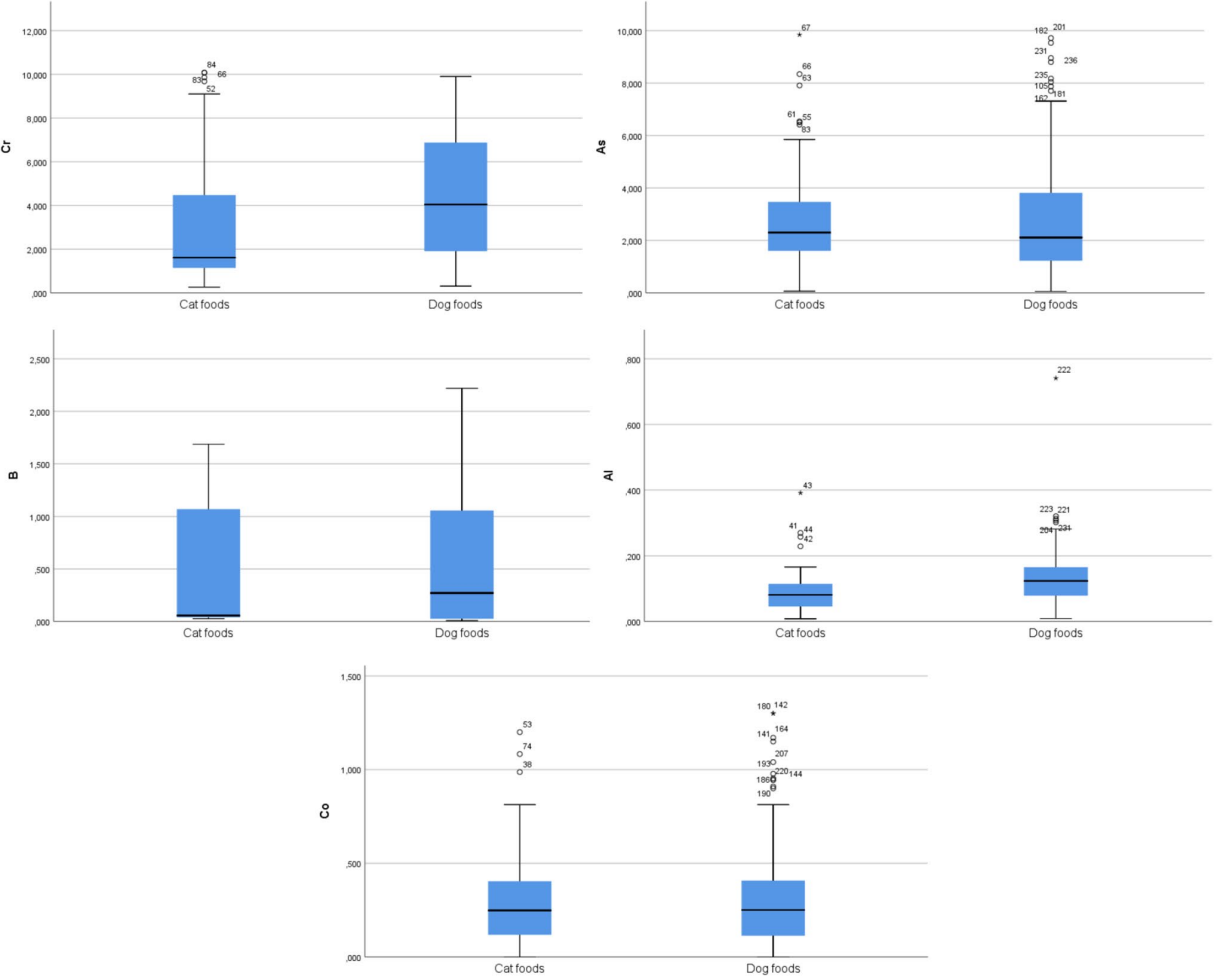
Box plot graphs show the mean Cr, As, B, Al, and Co levels in total cat and dog diet groups

Cr levels (mean ± SD) were 2.932 ± 2.665 µg/g and 4.422 ± 2.838 µg/g; As levels (mean ± SD) were 2.777 ± 1.931 µg/g and 2.785 ± 2.165 µg/g; B levels (mean ± SD) were 0.489 ± 0.600 µg/g and 0.558 ± 0.600 µg/g; Co levels (mean ± SD) were 0.299 ± 0.234 µg/g and 0.314 ± 0.280 µg/g for cat and dog diets, respectively. In the comparison of total dog and cat diets used in the study, no significant differences were observed between the groups in terms of mean As, B, and Co levels (*p* > 0.05). However, the mean Cr and Al levels in dog diets were statistically higher than those in cat diets (*p* < 0.001) (Table 1, Fig. 1).

**Table 1:** Cr, As, B, Al, and Co levels in total cat and dog diets

		**Cr (µg/g)**	**As (µg/g)**	**B (µg/g)**	**Al (µg/g)**	**Co (µg/g)**
**Cat diets (*n* = 84)**	Mean ± SD	2.932 ± 2.665	2.777 ± 1.931	0.489 ± 0.600	0.087 ± 0.061	0.299 ± 0.234
	Median	1.615	2.300	0.056	0.081	0.248
	Min–max	0.264–10.07	0.064–9.843	0.027–1.686	0.008–0.391	0–1.200
**Dog diets (*n* = 152)**	Mean ± SD	4.422 ± 2.838	2.785 ± 2.165	0.558 ± 0.600	0.127 ± 0.081	0.314 ± 0.28
	Median	4.042	2.108	0.271	0.123	0.250
	Min–max	0.309–9.900	0.056–9.717	0.008–2.219	0.009–0.741	0–1.300
	*p*^*1*^	< 0.001	0.486	0.105	< 0.001	0.906

^1^Mann-Whitney *U* test significance level for comparison of Cr, As, B, Al, and Co levels of cat and dog diet groups

**Fig. 2:**
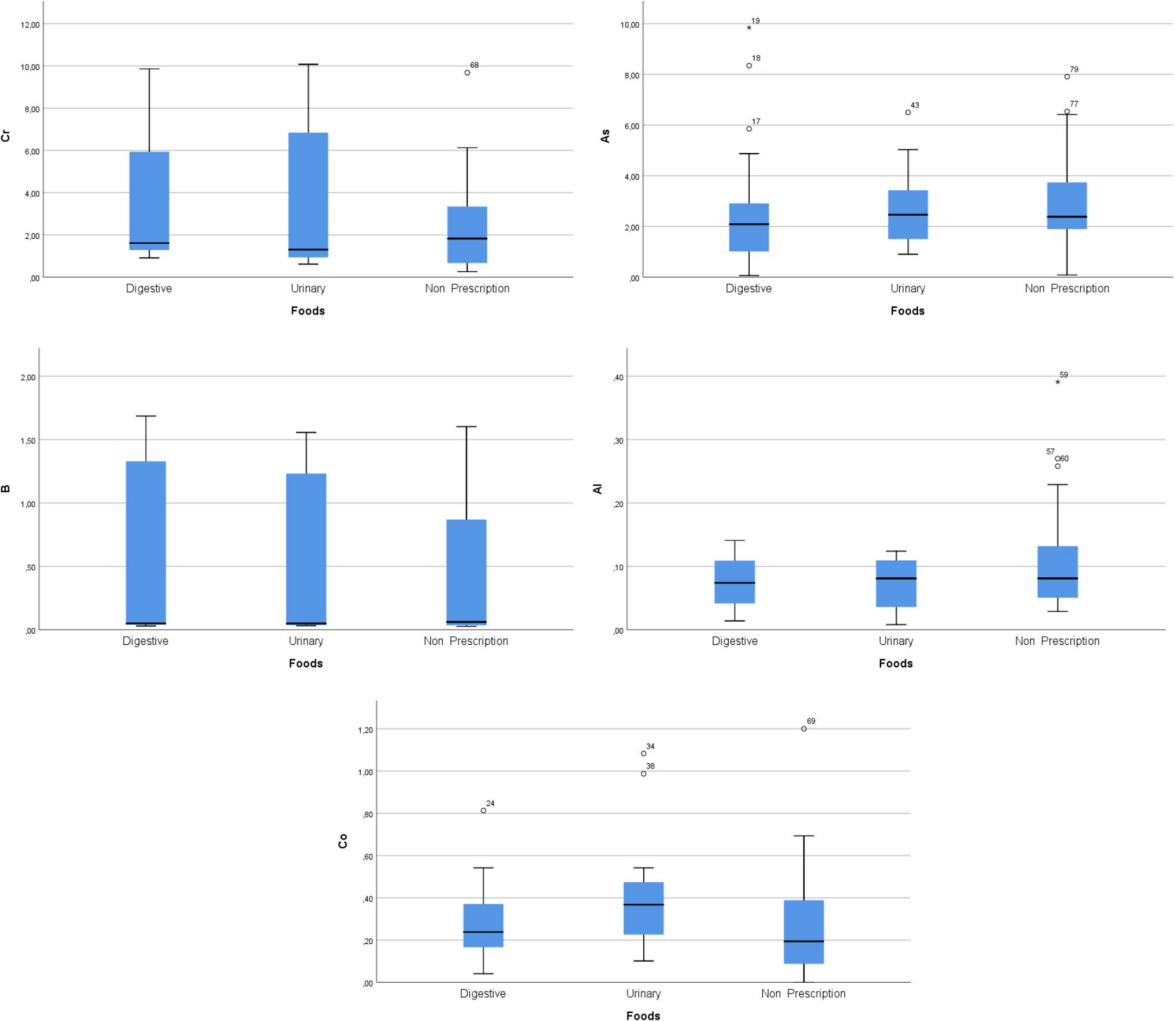
Box plot graphs show the mean Cr, As, B, Al, and Co levels in prescription and non-prescription cat diet groups

Among the prescription cat diets Cr, As, B, Al, and Co levels (mean ± SD) were measured as 3.277 ± 2.850 µg/g, 2.606 ± 2.453 µg/g, 0.511 ± 0.679 µg/g, and 0.285 ± 0.184 µg/g, respectively, for the digestive diet group; 3.599 ± 3.453 µg/g, 2.703 ± 1.500 µg/g, 0.558 ± 0.662 µg/g, and 0.405 ± 0.277 µg/g, respectively, for the urinary diet group. In the non-prescription cat diets Cr, As, B, Al, and Co levels (mean ± SD) were 2.391 ± 1.979 µg/g, 2.917 ± 1.803 µg/g, 0.441 ± 0.524 µg/g, 0.104 ± 0.076 µg/g, and 0.259 ± 0.235 µg/g, respectively. No statistically significant differences were observed between the groups (*p* > 0.05) (Table 2, Fig. 2).

**Table 2:** Cr, As, B, Al, and Co levels in digestive, urinary, and non-prescription cat diets

**Cat diets**		**Cr (µg/g)**	**As (µg/g)**	**B (µg/g)**	**Al (µg/g)**	**Co (µg/g)**
**Digestive (*n* = 24)**	Mean ± SD	3.277 ± 2.850	2.606 ± 2.453	0.511 ± 0.679	0.0727 ± 0.041	0.285 ± 0.184
	Median	1.615	2.087	0.049	0.074	0.238
	Min–max	0.910–9.840	0.064–9.840	0.030–1.690	0.010–0.140	0.040–0.810
**Urinary (*n* = 20)**	Mean ± SD	3.599 ± 3.453	2.703 ± 1.500	0.558 ± 0.662	0.073 ± 0.038	0.405 ± 0.277
	Median	1.308	2.463	0.049	0.081	0.367
	Min–max	0.062–10.08	0.910–6.500	0.030–1.560	0.010–0.120	0.10–1.080
**Non-prescription (*n* = 40)**	Mean ± SD	2.391 ± 1.979	2.917 ± 1.803	0.441 ± 0.524	0.104 ± 0.076	0.259 ± 0.235
	Median	1.828	2.382	0.061	0.081	0.194
	Min–max	0.264–9.678	0.086–7.908	0.027–1.603	0.029–0.391	0.000–1.200
	*p*^*1*^	0.363	0.352	0.923	0.236	0.093

^1^Kruskal-Wallis test significance level for comparison of Cr, As, B, Al, and Co levels for each cat diet group

**Fig. 3:**
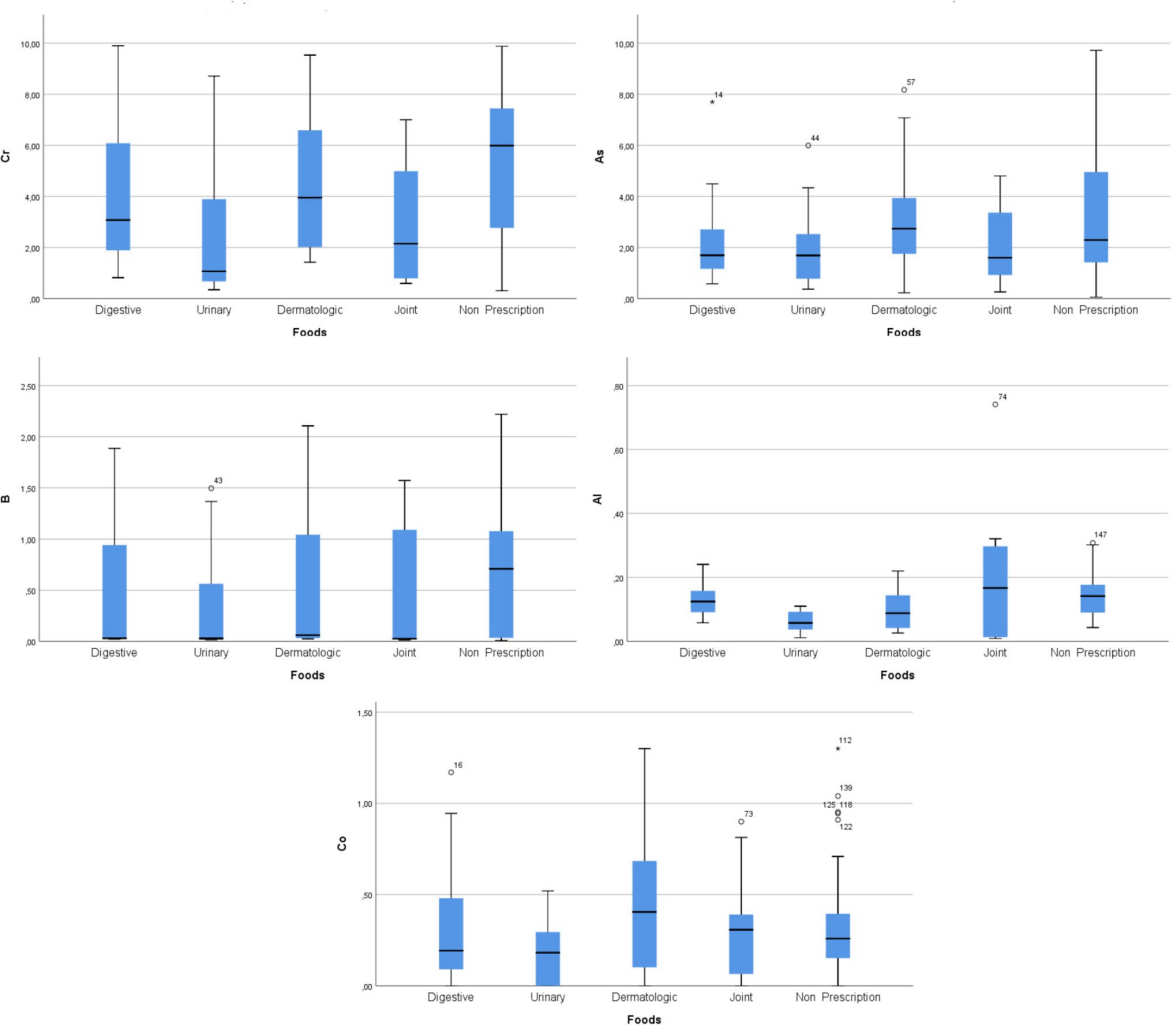
Box plot graphs show the mean Cr, As, B, Al, and Co levels in prescription and non-prescription dog diet groups

Among the prescription dog diets Cr, As, B, Al, and Co levels (mean ± SD) were measured as 4.063 ± 2.714 µg/g, 2.114 ± 1.491 µg/g, 0.503 ± 0.621 µg/g, 0.127 ± 0.043 µg/g, and 0.300 ± 0.293 µg/g, respectively, in digestive group; 2.455 ± 2.744 µg/g, 1.959 ± 1.520 µg/g, 0.338 ± 0.567 µg/g, 0.059 ± 0.032 µg/g, and 0.165 ± 0.170 µg/g, respectively, in urinary group; 4.531 ± 2.577 µg/g, 3.056 ± 2.084 µg/g, 0.529 ± 0.672 µg/g, 0.099 ± 0.059 µg/g, and 0.443 ± 0.390 µg/g, respectively, in hypoallergenic group; 2.974 ± 2.456 µg/g, 2.109 ± 1.530 µg/g, 0.451 ± 0.652 µg/g, 0.196 ± 0.208 µg/g, and 0.318 ± 0.292 µg/g, respectively, in joint group. In non-prescription dog diets Cr, As, B, Al, and Co levels (mean ± SD) were 5.169 ± 2.779 µg/g, 3.241 ± 2.473 µg/g, 0.650 ± 0.568 µg/g, 0.138 ± 0.055 µg/g, and 0.316 ± 0.246 µg/g, respectively. In the non-prescription diet, mean Cr levels were measured significantly higher compared to the urinary group (*p* < 0.001). Mean Al levels in the digestive, joint, and non-prescription groups were higher compared to the urinary group (*p* < 0.001). No statistically significant differences were observed for As, B, and Co between the groups (*p* > 0.05) (Table 3, Fig. 3).

**Table 3:** Cr, As, B, Al, and Co levels in prescription and non-prescription dog diets

**Dog diets**		**Cr (µg/g)**	**As (µg/g)**	**B (µg/g)**	**Al (µg/g)**	**Co (µg/g)**
**Digestive (*n* = 28)**	Mean ± SD	4.063^ab^ ± 2.714	2.114 ± 1.491	0.503 ± 0.621	0.127^b^ ± 0.043	0.300 ± 0.293
	Median	3.074	1.695	0.029	0.124	0.193
	Min–max	0.820–9.900	0.580–7.700	0.021–1.890	0.060–0.241	0.000–1.170
**Urinary (*n* = 16)**	Mean ± SD	2.455^a^ ± 2.744	1.959 ± 1.520	0.338 ± 0.567	0.059^a^ ± 0.032	0.165 ± 0.170
	Median	1.069	1.688	0.028	0.057	0.181
	Min–max	0.350–8.710	0.366–5.990	0.010–1.500	0.011–0.110	0.000–0.520
**Hypoallergenic (*n* = 20)**	Mean ± SD	4.531^ab^ ± 2.577	3.056 ± 2.084	0.529 ± 0.672	0.099^ab^ ± 0.059	0.443 ± 0.390
	Median	3.949	2.737	0.060	0.088	0.405
	Min–max	1.421–9.533	0.227–8.171	0.023–2.106	0.026–0.220	0.000–1.300
**Joint (*n* = 12)**	Mean ± SD	2.974^ab^ ± 2.456	2.109 ± 1.530	0.451 ± 0.652	0.196^b^ ± 0.208	0.318 ± 0.292
	Median	2.149	1.600	0.025	0.167	0.307
	Min–max	0.598–7.00	0.261–4.800	0.012–1.571	0.009–0.741	0.000–0.900
**Non-prescription (*n* = 76)**	Mean ± SD	5.169^b^ ± 2.779	3.241 ± 2.473	0.650 ± 0.568	0.138^b^ ± 0.055	0.316 ± 0.246
	Median	5.988	2.291	0.709	0.141	0.259
	Min–max	0.309–9.880	0.056–9.717	0.008–2.219	0.043–0.380	0.000–1.300
	*p*^*1*^	< 0.001	0.089	0.094	< 0.001	0.108

^1^Kruskal-Wallis test significance level for comparison of Cr, As, B, Al, and Co levels for each dog diet group. ^a,b^Different letters in the same column are statistically significant

The results showed that the mean Cr levels were significantly higher in the non-prescription dog diets compared to the non-prescription cat diets (*p* < 0.001). The mean B levels were higher in digestive and urinary cat diets compared to dog diets (*p* < 0.05). Mean Al levels were higher in the digestive and the non-prescription dog diets compared to the digestive and the non-prescription cat diets (*p* < 0.001). Mean Co levels were higher in the urinary cat diet group compared to the similar dog diet group (*p* < 0.01) (Table 4).

**Table 4:** *p* values for the comparison of various cat and dog diets

Cat x dog diets	Cr	As	B	Al	Co
Digestive (*n* = 52)	0.099	0.633	0.046	< 0.001	0.583
Urinary (*n* = 36)	0.158	0.077	0.012	0.189	0.004
Non-prescription (*n* = 116)	< 0.001	0.832	0.455	< 0.001	0.151

## Discussion

One of the objectives of this study was to highlight the PTEs in commercial dry pet diets, which play a significant role in cat and dog nutrition and are widely available in the global market. Therefore, in this study, both non-prescription commercial dry pet diets recommended for healthy cats and dogs and prescription diets suggested as supportive treatment for various diseases were evaluated separately.

When the compositions of prescription and non-prescription pet diets from different brands and formulations were compared in terms of toxic elements, it was notably observed that the mean Cr and Al levels differed significantly between cat and dog diets. Cr content in pet food may be influenced by the inclusion of animal protein sources such as fish, poultry, and red meat. The extent to which tissues and organs known to accumulate high levels of copper—such as the heart, lungs, liver, and kidneys—are used as protein sources may also contribute to differences in concentrations among pet foods [22]. Although trivalent Cr was suggested to be an essential element, recent studies indicate that Cr should be regarded as a pharmacologically active rather than essential [23–25]. Additionally, the European Food Safety Authority (EFSA) has concluded that Cr is not an essential element for humans or animals [26]. According to European Pet Diet Industry Federation (FEDIAF) nutritional guidelines, if chromium oxide is utilized, about 0.25% of high-purity chromium (III) oxide (Cr2O_3_), devoid of soluble Cr, should be incorporated into the feed [27]. Although low-dose Cr intake has been shown to have positive health effects, such as stimulating enzymatic and non-enzymatic antioxidant responses and acting as a glucose tolerance factor [28–30], higher concentrations are known to have toxic effects [31]. Although the maximum tolerable limits (MTL) of Cr as an additive for large animal feed were determined by the Association of American Feed Control Officials (AAFCO), minimum and maximum limits for cats and dogs have not been established by either FEDIAF or AAFCO [27, 32]. However, according to the United States Food and Drug Administration (FDA), the estimated MTL for Cr was reported as 10 µg/g dry matter [33]. In this study Cr levels in all cat and dog diet groups were below the MTL determined by FDA. However, numerically the highest mean Cr level in dog diet was observed in non-prescription diets, while the lowest was found in urinary group. Among all cat diets, numerically the lowest mean Cr level was detected in non-prescription diets, whereas similar levels were observed in the urinary and digestive diet groups. None of the pet diet products used in the study specified the presence of Cr or its compounds among their ingredients. Therefore, the measured Cr levels in the pet diets may result from the plant- and/or animal-based ingredients present in their formulation. In particular, the lower Cr levels detected in the urinary dog diet group, which is used in the management of urinary system diseases such as renal failure, compared to non-prescription dog diets suggest a possible association with the protein source (poultry, fish, or red meat products). Similar findings obtained from dog diets could have been expected in cat diets as well; however, the Cr levels in all cat diet groups appeared to be lower than those in dog diets. In a study investigating the Cr levels in various ingredients such as ground rice, corn grain, poultry viscera meal, and beef meat, mean Cr results were 300 g/kg, 170 g/kg, 150 g/kg, and 100 g/kg, respectively [28]. Zafalon et al. (2021) reported that the mean Cr levels in dog diets were 4.73 (0.90–7.74) µg/g; in cat diets were 3.90 (0.87–10.22) µg/g [17]. In a similar study, mean Cr levels in both cat and dog diets were 2.13 μg/g (0.58–3.73) [34]. These findings were consistent with our results and remained below the MTL, indicating no potential risk of dry diet-related Cr toxicosis in both dogs and cats.

As is considered as PTEs in both humans and animals. As exposure in cats and dogs may occur due to environmental contaminants or dietary sources. A study reported that detectable levels of As (0.86–12.50 µg/g) were found exclusively in pet diets containing fish or fish derivatives, suggesting possible contamination within the aquatic ecosystem. A recent similar study showed that the mean As concentration was 0.159 µg/g in premium dog diets and 0.172 µg/g in premium cat diets and was consistent with the previous report of Squadrone et al. (2017) [35], As levels were significantly higher in fish-based feeds [6]. In another study, the median As concentration in fish-based dry dog diets was 0.343 mg/Mcal (0.025–1.104 mg/Mcal), which was significantly higher than poultry-based and red meat-based formulations [36]. These findings highlight both aquatic food chain contamination and the risk of toxicosis in pet diets containing fish products. In our study, groups were not categorized according to protein source. Further studies are needed to support these findings. According to FDA, MTL for As was determined as 12.5 µg/g for dog and cat diet products [33]. In a similar study, As was not detected in commercial cat and dog diets [17]. In our study, As was detected in all diet groups and the results were statistically similar between all prescription and non-prescription diet types. In all dog diet groups, numerically, the highest mean As level was measured in non-prescription and the lowest in protein-restricted urinary diets. Although all results were below the MTL determined by the FDA, the cumulative nature of As suggests that detectable levels may contribute to chronic diet-associated toxicosis in both cats and dogs.

B has not been considered an essential nutrient for humans and animals due to insufficient characterization of its biological role. However, studies have reported its significant roles in the physiology and function of various tissues and organs including symbiotic and prebiotic involvement [37], carbohydrate and lipid metabolism [38], neuroprotective activity [39, 40], and redox balance [41]. Although various studies have examined the effects of B intake in human medicine and experimental research [42–45], the literature on its significance and efficacy in cats and dogs remains limited. Although B toxicity is particularly significant in various plant species [46–48], it has also been reported reported in humans and animals via inhalation, dermal, and oral exposure [49]. To the authors’ knowledge, B toxicity in cats and dogs has not been reported in the literature. Although the lethal dose of B in cats and dogs remains unclear, one study reported that a high intake of boric acid (1750 µg/g) resulted in testicular atrophy and a moderate reduction in sperm production, whereas a B concentration of 350 ppm was well tolerated by dogs [50]. In a recent study, MTL for B in cats and dogs was suggested to be 150 µg/g based on the reference value of the National Research Council (2005) [17]. The B levels in our study are remarkably lower than the values reported in the referenced studies. Considering the reports of Weir and Fisher (1972) [50 and Zafalon et al. (2021) [17], which determined the minimum and maximum B levels, the levels measured in cat (0.027–1.686 µg/g) and dog (0.008–2.219 µg/g) diets in this study were below the toxic threshold. Therefore, it was concluded that they did not pose a potential risk of toxicosis in cats and dogs. However, studies on the toxicology and metabolism of B in dogs and cats are limited.

Al is primarily excreted by the kidneys and tends to accumulate in tissues and organs at toxic concentrations, potentially leading to various clinical manifestations such as muscle twitching, convulsions, tetraparesis, and coma [51]. However, the intestine is considered a protective barrier against the toxicity of orally ingested Al, as its absorption is very low due to its high insolubility [52]. Based on various studies examining the intake rates of Al compounds through diet, the European Food Safety Authority (EFSA) has established the Tolerable Weekly Intake (TWI) of Al in dogs at 1 µg/g/week [53]. However, MTL of Al has not been reported for cats. In a study, the mean Al levels in premium and non-premium dog diets were 0.089 and 0.078 µg/g, respectively. In premium and non-premium cat diets, they were 0.081 and 0.072 µg/g, respectively [6]. In another study, the minimum and maximum detected Al concentrations (µg/g dry matter) were 0–2406 in 72 dog diets and 0–582 in 28 cat diets, reflecting wide ranges [17]. According to our results, Al levels were relatively higher in dog diets than in cat diets. Al levels may vary depending on the use of additives, clays, or mineral supplements, as well as possible contamination during processing or packaging. Plant-based ingredients, particularly cereals and grains, can contain higher levels of Al due to uptake from soil or Al-based fertilizers. Ingredients such as rice, wheat, and gluten which are prevalent in cat diets may contribute to higher Al concentrations. In a study investigating Al levels in hair, the mean Al concentration was significantly higher in dog hair (136.66 µg/g) than in cat hair (94.31 µg/g). In the same study, it was emphasized that animals living outdoors had higher Al levels than those living indoors [54]. This finding, along with the low Al measurements obtained in our study, suggests that Al exposure in cats and dogs may be more closely related to environmental contaminants than to pet diets. The mean Al level in dog diets was higher in digestive, joint, and non-prescription groups compared to the urinary group. Although no Al compounds are specifically listed in commercial pet diets, the source of Al in these products may originate from additives containing certain Al compounds [51] or may be associated with the protein source included in the formulation [18]. In our study, lower Al levels were observed in prescription diets formulated with restricted protein content for urinary system diseases, particularly kidney diseases. Similarly, although not statistically significant, the lower Al concentrations in hypoallergenic diets compared to other groups may be associated with the use of processed (hydrolyzed) protein sources and technological additives containing lower Al components.

The intestinal microbiota of dogs and cats can produce cobalamin (vitamin B_12_) in the presence of Co. However, since the site of cobalamin production is distal to its site of absorption, this process appears to provide little benefit to the animal [55]. Nevertheless, since many commercial pet diets are supplemented with cobalamin, Co may be considered an undesirable metal in pet diets due to its potential toxic effects on thyroid, heart, nervous, and hematopoietic system in higher doses [15, 56]. Adequate Co levels are particularly important for ruminants, whereas Co toxicosis has not commonly observed in cats and dogs. According to a FDA report (2011) [33], the MTL for Co in cat and dog diets is 2.5 µg/g. In all diet groups, mean Co levels in our study were below this threshold. The higher mean Co levels in the urinary diet group of cats compared to dogs appear to be clinically insignificant, as both concentrations were below the MTL and posed a low risk of toxicity. According to the study by Zafalon et al. (2021) [17], the mean Co level in dog diets was 1.65 µg/g (0–14.11) and in cat diets was 0.66 µg/g (0–2.49). Our findings are consistent with those of the previous study and do not indicate a potential risk of diet-related Co toxicosis.

In a recent study conducted in the United Arab Emirates evaluating various metal concentrations in 26 different dry cat food products, it was reported that the concentrations of Al and Co exceeded the MTL set by FEDIAF in two samples (7.69%). Cr concentrations, on the other hand, were found to be in compliance with regulatory safety standards, consistent with the findings of our study. The study also emphasized the relationship between the variation in measured elemental concentrations and the protein source in the pet food composition [57]. In another recent study conducted in South Africa, the mean concentrations of As and Co detected in various dog food samples were reported as 7.47 mg/kg and 4.01 mg/kg, respectively, while in cat food samples, the concentrations were 2.92 mg/kg for As and 2.44 mg/kg for Co. Although the As levels remained below the MTL, it was reported that Co concentrations exceeded the MTL allowed by the FDA in 10 dog food brands (32.2%) and 7 cat food brands (22.5%) [58]. In contrast to these findings, all premium dry dog and cat foods analyzed in our study contained Co concentrations within acceptable limits. Notably, while previous studies have reported Co levels exceeding safety thresholds in a substantial proportion of both dog and cat foods, our results indicate a more favorable profile among premium brands. Furthermore, statistical analysis revealed no statistically significant difference (*p* > 0.05) in Co concentrations between premium dog and cat foods in our dataset, supporting the conclusion that Co levels in these products remain consistently within regulatory limits. Considering the results of numerous studies from different regions, the significant differences in the obtained metal contents highlight the potential role of regional and compositional variability in the heavy metal contamination of commercial pet foods. Additionally, the deviations observed between different studies emphasize the importance of continuous, region-specific monitoring, as well as the potential impact of formulation practices and raw material quality on the heavy metal content.

The limitations of this study include the small number of different types of prescription cat diet groups and the relatively low sample size in prescription dog diet groups although statistically sufficient. Additionally, we believe that future studies evaluating diet groups based on their protein sources would yield highly valuable results.

## Conclusion

The concentrations of Cr, As, B, Al, and Co in prescription and non-prescription commercial dry diets for cats and dogs were below the MTL established by regulatory authorities, indicating that these PTEs do not pose a risk of diet-related toxicosis in cats and dogs. However, given the potential for regional and formulation-based variability in heavy metal contamination, these findings underscore the need for regular monitoring and quality control in the production of pet foods to ensure long-term safety and compliance with nutritional and toxicological standards.

## Publisher’s Note

Springer Nature remains neutral with regard to jurisdictional claims in published maps and institutional affiliations.

## Data Availability

No datasets were generated or analysed during the current study.
